# Quintuplet pregnancy: a case report from Pakistan

**DOI:** 10.1097/MS9.0000000000002598

**Published:** 2024-09-24

**Authors:** Sardar Noman Qayyum, Eman Alamgir, Iqra Alamgir, Gulmeena Aziz Khan, Unsa Alamgir, Muhammad Rehan, Samim Noori

**Affiliations:** a Bacha Khan Medical College, Mardan; b University Medical and Dental College, Faisalabad; c Al-Nafees Medical College and Hospital, Islamabad, Pakistan; d Faculty of Medicine, Nangarhar University, Nangarhar, Afghanistan

**Keywords:** clomiphene citrate, neonatal mortality, preterm birth, quintuplet pregnancy

## Abstract

**Introduction and importance:**

Quintuplet pregnancies are exceptionally rare outcomes of fertility treatments, that is clomiphene citrate therapy. These high-order multiple pregnancies carry significant risks for both maternal and neonatal health, necessitating specialized care to manage complications effectively.

**Case presentation:**

A 26-year-old woman, gravida three para two, presented with preterm labor at 32 weeks gestation following clomiphene citrate self-medication. Diagnosed with quintuplets, she underwent an emergency cesarean section at Allied-1 Hospital, Faisalabad, delivering five neonates alive. Unfortunately, all five neonates succumbed to death due to perinatal asphyxia, three within 24 h and two on the third day.

**Clinical discussion:**

Higher-order multiple pregnancies, such as quintuplets, are associated with increased risks of obstetric complications, including pre-eclampsia, gestational diabetes, and fetal growth restriction. Preterm birth further exacerbates the risk of neonatal complications like respiratory distress syndrome, perinatal asphyxia, and death. The patient’s care highlights the challenges of managing such pregnancies in resource-limited settings, emphasizing the need for specialized antenatal and neonatal care facilities.

**Conclusion:**

This case highlights the critical need for advanced medical care in managing high-order multiple pregnancies. The outcomes reflect the significant challenges posed by preterm labor and neonatal complications in quintuplet pregnancies. There is the need for enhanced antenatal care, healthcare infrastructure, and multidisciplinary teams for the efficient management of high-order multiple pregnancies in Pakistan, where neonatal and maternal mortality rates are surprisingly high.

## Introduction

Highlights

- A 26-year-old woman conceived quintuplets following clomiphene citrate self-medication, and presented with preterm labor at 32 weeks gestation, and underwent an emergency cesarean section.
- Unfortunately, all five neonates succumbed to death due to perinatal asphyxia.
- The case highlights the maternal and neonatal risk associated with higher-order multiple pregnancies and emphasizes the need for close monitoring and specialized care.
- It also discusses the poor state of neonatal care in Pakistan as reflected by high neonatal and maternal mortality rates.
- It also spotlights the importance of family planning and patient education in preventing self-medication of prescription-only drugs, and enhancing patients’ commitment to regular medical checkups during pregnancy.

Quintuplet pregnancy, the conception of five offsprings in the same gestation, is an exceptionally rare outcome of ovulation induction therapies^1^. The global incidence of quintuplet pregnancies is extremely rare, that is 1 in about 55 million births. Such pregnancies pose a significant challenge for both the mother and the neonates. The mother faces increased risks of complications such as preterm labor, pre-eclampsia, and gestational diabetes, along with the physical strain and emotional strain^2^. Preterm birth is almost inevitable, with an average gestation period of 29 weeks^3^ leading to low birth weight, underdeveloped organs, and respiratory issues, which often necessitate prolonged stays in a Neonatal Intensive Care Unit (NICU). Ovarian hyperstimulation by Clomiphene citrate results in more than 60% of the multifetal pregnancy cases^1^. Clomiphene citrate is a selective estrogen receptor modulator (SERM) that works by blocking estrogen receptors in the hypothalamus, leading the body to falsely perceive low estrogen levels. This triggers the hypothalamus to release more gonadotropin-releasing hormone (GnRH), which causes increased release of follicle-stimulating hormone (FSH) and luteinizing hormone (LH), ultimately leading to maturation of ovarian follicles, and ovulation. Following clomiphene citrate therapy, there is a 0.13% risk for quintuplets, a 0.3% risk for quadruplets, a 0.3–0.5% risk for triplets, and a 9% risk for twins^4,5^. Moreover, higher-order multiple pregnancies not only increases risks for maternal and fetal complications but also increases the risks for psychosocial issues such as emotional stress, depression, impaired relationships, economic burden, etc.

In this case report, we report a case of quintuplet pregnancy conceived with clomiphene citrate, presented at a tertiary care hospital’s emergency at 32nd week of gestation. It signifies the lack of family planning, inadequacy of the healthcare system, which led to the patient’s emergency arrival due to preterm labor. It also highlights the risks associated with multifetal pregnancy, lack of rigorous prenatal monitoring, compromised diagnostic quality, and challenges associated with advanced neonatal care. This work has been reported in line with the Surgical CAse Report (SCARE) 2023 guidelines^6^.

### Case presentation

A 26-year-old pregnant woman, gravida three para two, presented with diffuse lower abdominal pain and early preterm labor to the emergency department of Allied-1-hospital in Faisalabad. The patient had undergone lower segment cesarean section twice in previous pregnancies and had two girls alive and healthy, one aged 5 and the other 7. The patient was a known case of quadruplet pregnancy and initially visited a secondary healthcare facility but due to the nonavailability of the ICU and neonatal intensive care units (NICU), she was referred to the nearest tertiary healthcare facility.

The patient had no history of smoking, and the current pregnancy was conceived with clomiphene citrate, 50 mg. The patient told doctors that she could not conceive a pregnancy after the birth of her second child for 4 years, so she took clomiphene citrate by herself to get pregnant without any medical checkup or doctor’s advice. The current pregnancy was confirmed with a urine pregnancy test (UPT).

The first trimester passed uneventfully. During the second trimester, she felt quickening for the first time, and her blood pressure began to rise moderately due to pregnancy-induced hypertension, for which she consulted a local clinic for the first time. An ultrasound was performed, which revealed quadruples, and the patient was diagnosed as a case of quadruplet pregnancy. The doctor also suspected the risk of preterm birth and advised McDonald’s cerclage. The McDonald’s cerclage was performed by an attending gynecologist at the local clinic to reduce the risk of preterm labor after the detection of multifetal pregnancy. Multifetal reduction was not offered to the patient at this stage due to the unavailability of this procedure and the absence of legal legislation permitting the procedure in Pakistan.

### General physical and pelvic examinations

Upon examination, the patient’s vital signs were relatively normal except for mildly elevated blood pressure, that is 130/90 mmHg. Physical examination revealed pallor. The pelvic examination showed that the cervix was 4 cm in length, soft, and centrally positioned. The cervix had undergone 40% effacement indicating early stages of labor. However, the amniotic membranes surrounding the fetuses were intact. Further assessment revealed that the patient’s abdomen was soft to the touch, and the symphysio-fundal height was 42 cm.

### Investigations

The first ultrasound, performed during the second trimester, revealed tetra-amniotic tetra-chorionic quadruplets with variable lie and presentation. The placenta was located in the anterior and fundo-posterior uterus wall. The fetal movements and fetal cardiac activity were normal in all four fetuses, and no gross abnormality was found. The cervical length was 4 cm. A follow-up ultrasound scan at the 29th week of gestation revealed increased resistance in the umbilical arteries of each fetus, with the systolic/diastolic (S/D) ratio elevated up to 4.7. Additionally, the amniotic fluid was found to be at the lower limit, with an amniotic fluid index (AFI) less than 8 cm. This ultrasound scan also determined the position and orientation of each fetus. Fetus 1 was in cephalic presentation, while fetus 2 was in breech presentation. Fetuses 3 and 4 were in transverse presentation. Fetal measurements obtained are given in Table 1.

**Table 1:** Fetal measurements (average) as shown by ultrasound scans performed at 17th and 29th gestation weeks

Parameter (s)	Value(s) at 17th week	Value(s) at 29th week	50th percentile at 17th week	50th percentile at 29th week
Biparietal diameter (BPD)	3.7 cm	7.2 cm	3.6 cm	7.4 cm
Femur length (FL)	2.2 cm	5.8 cm	2.2 cm	5.4 cm
Head circumference (HC)	13.1 cm	26.2 cm	13.5 cm	27.3 cm
Umbilical cord	Three vessels	Three vessels	—	—
Fetal weight	171 g	1430 g	181 g	1379 g
Abdominal circumference	—	24.8 cm	—	25 cm

At the 32nd week of gestation, another ultrasound scan and biophysical profile was performed when the patient was brought at the emergency department of Allied hospital, which revealed the presence of all five fetuses, each with a separate amniotic sac and chorion. Baseline labs at the 17th, 29th, and 32nd gestation weeks revealed elevated WBC count, urine albumin, and uric acid levels.

Table 2 summarizes the baseline labs.

**Table 2:** Baseline labs performed at 17th, 29th, and 32nd gestation weeks

Parameter (s)	Value(s) at 17th week	Value(s) at 29th week	Value(s) at 32nd week	Reference
Hemoglobin	10.8	10.7	11.8 after transfusion	12–17 g/dl
White blood cells	12.5	—	19.7	4.5–11×10^9^/l
Platelet count	231 000	—	175 000	150 000–400 000 µl
Neutrophils	69	—	86	50–70%
Lymphocytes	27	—	12	25–35%
Monocytes	03	—	01	4–6%
Eosinophils	01	—	01	1–3%
Prothrombin time (PT)	—	15	15	15 s
Activated partial thromboplastin time (APPT)	—	34	35	35 s
Blood glucose (random)	114	—	80	<140 mg/dl
Blood group	A+ve	—	—	—
Packed cell volume (PCV)	30.7	—	—	35.5–44.9%
MCV	86.7	—	—	75–95 fl
MCH	29.1	—	—	27–32 pgm

### Treatment

The patient initially received symptomatic treatment with steroids, micronized progesterone, and pain relievers at a secondary healthcare facility and was referred to Allied-1-hospital, Faisalabad. The final assessment at the Allied-1-hospital revealed a case of quintuplet pregnancy. The patient was counseled for emergency cesarean section and the potential complications associated with multiple pregnancies. Informed consent was obtained for cesarean section and postpartum intrauterine contraception before preparing the patient for cesarean section. She also received 473 cm^3^ of whole blood which raised her Hb from 10.7 to 11.8 g/dl. The ICU and NICU teams were well-prepared to ensure smooth procedures. An emergency cesarean section was performed by a consultant gynecologist under spinal anesthesia. During the surgery, dense adhesions were found between the rectus sheath and rectus muscles, along with evidence of previous scar dehiscence. The bilateral adnexa was found to be healthy. Five neonates were delivered alive along with their placenta and membranes. The cervical cerclage was removed and a postpartum contraceptive device (PPIUCD) was implanted in the uterus. The details of the five neonates are as follows:

First neonate; a female with cephalic presentation weighed 1.1 kg, APGAR score of 5/10 and 7/10 at 1 and 5 min, respectively.

The second neonate; a male with breech presentation weighed 1.6 kg, APGAR score of 5/10 and 6/10 at 1 and 5 min, respectively.

The third neonate; a male with cephalic presentation weighed 1.8 kg, APGAR score of 6/10 and 8/10 at 1 and 5 min, respectively.

The fourth neonate; a female with cephalic presentation weighed 1.6 kg, APGAR score of 7/10 and 8/10 at 1 and 5 min, respectively.

**Figure 1:**
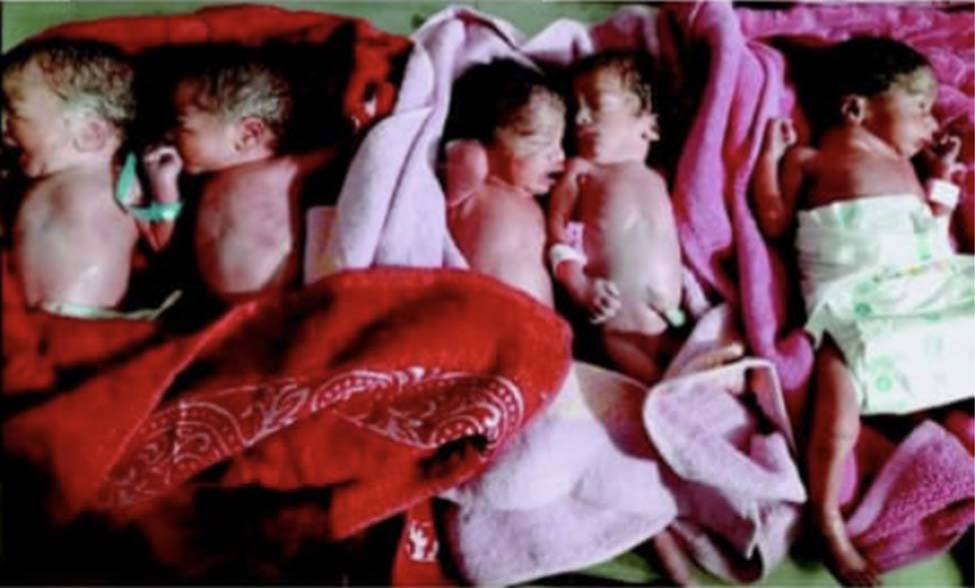
Five neonates, three females, and two males, right after birth.

The fifth neonate; a female with breech presentation weighed 1.3 kg, APGAR score of 7/10 and 9/10 at 1 and 5 min, respectively. Figure 1 shows the quintuplets on the first day right after birth in the NICU.

### Follow-up

The mother remained physically healthy during the postpartum follow-up and was discharged after a week. All neonates exhibited cyanosis at birth but had normal limb and genital development. They were immediately placed in incubators, and blue light therapy was initiated. Despite these interventions, three of the five infants succumbed to perinatal asphyxia within 24 h. The remaining two neonates also passed away on the third day due to the same condition. The mother was profoundly affected by the loss of all five of her children. Even months after the incident, the mother has not recovered psychologically from the trauma.

### Patient’s perspective

**Figure 2:**
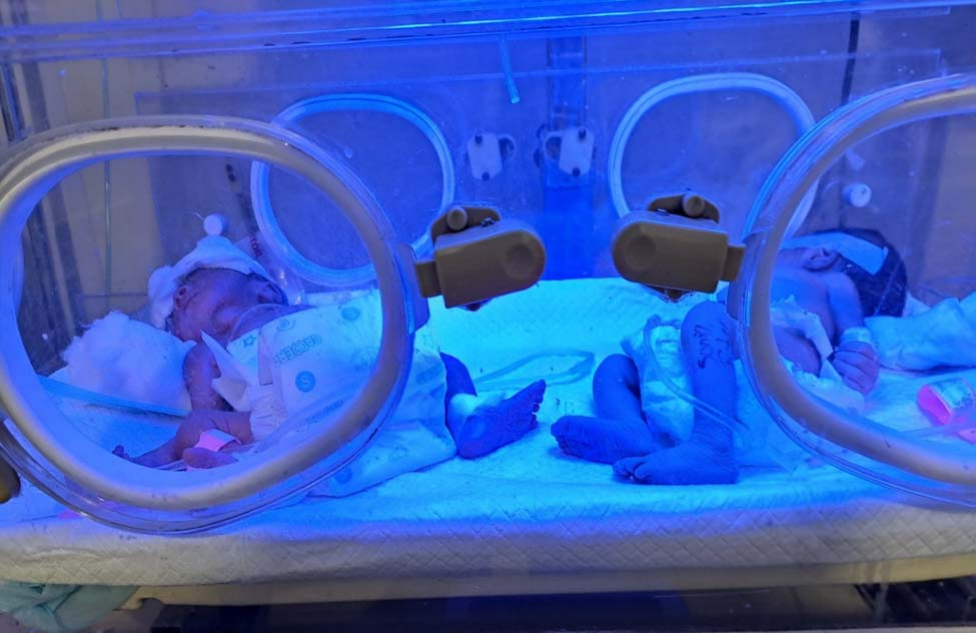
Two neonates in incubators in the Neonatal Intensive care unit (NICU) on day 2.

The mother was affected by the loss of all five of her children and reported inadequate neonatal care for their deaths. She struggled emotionally and psychologically, showing significant signs of distress. Although psychiatric support was offered, she declined, reflecting the ongoing stigma surrounding mental healthcare in developing countries. In Figure 2, we have shown two surviving neonates on day 2, which also died later.

## Discussion

Clomiphene citrate is a selective estrogen receptor modulator given to women with WHO group II ovulatory dysfunction^1^. Medications used to treat infertility often promote the maturation of multiple eggs, increasing the incidence of multiple pregnancies. Therefore, uterine distention can be expected with higher-order multiple pregnancies with clomiphene citrate treatment and can results in premature birth and other associated complications^7^. Evidence suggests that more than 70% of women ovulate and 36% conceive pregnancy following the treatment. To avoid multifetal pregnancies, low-dose clomiphene citrate is prescribed along with regular ultrasounds and close monitoring of estradiol levels^1^.

This is a prescription drug, but in this case, the patient took it without the doctor’s advice. The patient reported experiencing significant family pressure due to her inability to conceive for four years, despite her efforts. Although she already had two healthy daughters, societal expectations for a son, coupled with her illiteracy, made her to use clomiphene citrate. During the 4 years of trying to conceive, her menstrual cycles were normal, and no definitive cause of infertility was reported. However, it is important to note that she did not consult any gynecologists or doctor during this period, so a definitive assessment of her fertility status could not be made. The availability of prescription-only drugs to patients without a proper prescription remains a significant concern for improving healthcare infrastructure.

Quintuplet pregnancy is one of the exceptionally rare outcomes of fertility treatment and occurs due to the development of multiple follicles, which can be influenced by dose variability and adjunctive treatment. Higher-order multiple births are associated with obstetric complications and higher incidences of perinatal mortality. Thus, women with multifetal pregnancies require special antenatal care by a multidisciplinary team, including an obstetrician, gynecologist, dietician, neonatologist, general physician, and psychiatrist for early detection and management of complications to improve the perinatal outcomes^8^. Unfortunately, in Pakistan, such an approach to the patient’s care is not available, as reflected by higher neonatal mortality rates. Due to the poor quality of diagnostic assessments at local clinics, one or two fetuses can go undetected in early pregnancy ultrasounds. In this case, the patient was initially diagnosed with a quadruplet pregnancy, but a subsequent evaluation at a tertiary healthcare facility revealed a quintuplet pregnancy.

The average gestation period for quintuplet pregnancy is 28–29 weeks^3^. Infants born prematurely, 11 weeks before the normal gestation period, are at higher risk of perinatal complications, including respiratory distress syndrome, bronchopulmonary dysplasia, perinatal asphyxia, apnea, sepsis, patent ductus arteriosus (PDA), intraventricular hemorrhage, necrotizing enterocolitis, hypothermia, hypoglycemia, hyperbilirubinemia, and death^9^. In this case, all prematurely born neonates succumbed to death due to perinatal asphyxia. Mothers with multifetal pregnancies are also more likely to be diagnosed with pre-eclampsia, preterm premature membrane rupture, and shock due to excessive bleeding^2^. Therefore, women with higher-order multiple pregnancies should be referred to an adequate healthcare facility early during the pregnancy. Selective fetal reduction is usually performed in the early pregnancy, that is the first trimester, when a multifetal pregnancy presents early with higher risks of complications such as genetic or structural abnormalities in one or more fetuses, uterine anomalies, etc. The procedure involves the identification of the fetus using ultrasound and injecting potassium chloride (KCL) into the fluid-filled pouch of the fetus to stop the fetal heart. It has a success rate of 99.5–100% with minimal maternal complications^10,11^. The practice of fetal reduction is influenced by geographic and ethnic factors and the removal of one or more defective fetuses is deemed legal in Islamic religion^12^. However, fetal reduction was not advised by the doctors in this case due to the unavailability of the procedure, the absence of an experienced practitioner, and lack of legal legislation on selective fetal reduction in Pakistan. The antenatal care in Pakistan is a tragedy itself, as indicated by the elevated neonatal mortality rate (NMR) and maternal mortality rate (MMR). The NMR in Pakistan, ranging from 41 to 49.4 deaths/1000 live births^13^, is one of the highest in South Asia and accounts for ~7% of all newborn deaths globally. A significant disparity exists between rural and urban areas, with rural facilities experiencing a substantially higher NMR (62 deaths/1000 live births)^14^ than urban facilities (47 deaths/1000 live births)^14^, reflecting constraints faced by rural women in accessing adequate prenatal services crucial for mitigating risks of complications including newborn deaths. The MMR is 186 deaths per 100 000 live births^15^, with Pakistan ranking 122nd among 190 countries in the WHO performance report. Pakistan has to reduce the current NMR by 11.6% annually to achieve the sustainable development goal of 12 or fewer neonatal deaths per 1000 live births by 2030^16^. The government also has to take measures to achieve the global maternal mortality of 70 or fewer maternal deaths per 100 000 live births^15^.

To our knowledge, this is the first case of quintuplet pregnancy following clomiphene citrate therapy. A mother, in 2008, delivered five healthy infants via spontaneous labor without any history of fertility treatment, unlike our case. Both mother and infants were healthy and discharged after a 4-day follow-up. Another case of quintuplets was reported, in which a mother gave birth to three boys and two girls in Karachi via cesarean section^17^. In another case, a patient conceived spontaneously quintuplet pregnancy in Somalia, and a cesarean section was performed. Unfortunately, one of the five neonates died, and the rest spent 5 weeks in NICU and were discharged later^8^.

Through this case, we want to highlight that quintuplet pregnancies, though extremely rare, come with significant risks for both the mother and the neonates, and the fertility treatments like clomiphene citrate should be used under strict medical supervision. Effective family planning and patient education can prevent unintended high-order multiple pregnancies. Educating patients can prevent the use of fertility medications without proper prescription and doctors’ consultation. This case also highlights the importance of the accessibility of advanced diagnostic tools in resource-limited areas and patient counseling. It can help with early screening and enhance patients’ commitment to regular checkups. The management of multifetal pregnancies requires a multidisciplinary approach, including obstetricians, gynecologists, dieticians, neonatologists, and psychiatrist.

## Conclusion

We report a case of quintuplet pregnancy following self-medication of clomiphene citrate and highlight the significant obstetric and neonatal complications associated with higher-order multiple gestations. In this case, all five neonates succumbed to death due to perinatal asphyxia, while the mother remained physically healthy. Longer follow-up revealed that the mother was emotionally and psychosocially disturbed due to the loss of her five infants.

Quintuplet pregnancy can be managed by a multidisciplinary team, including a gynecologist, neonatologist, psychiatrist, dietician, etc. Patient education, counseling, and family planning are important to avoid self-medication, to enhance patient’s commitment to regular checkups and early screening of associated complications. Multifetal reduction can be performed, but there is a need for the training of professionals for the procedure and legal legislation permitting the procedure in the country.

## Ethical approval

Ethical approval was not needed for writing a case report in our settings.

## Consent

Written informed consent was obtained from the patient for publication of this case report and accompanying images. A copy of the written consent is available for review by the Editor-in-Chief of this journal on request.

## Source of funding

The authors received no funding from any individual or institution, and this work is completely voluntary.

## Author contribution

S.N.Q.: supervision, writing initial draft, reviewing, and revision; E.A.: data curation and writing initial draft; I.A.: writing initial draft and reviewing; G.A.K.: writing and reviewing; U.A.: data curation and writing initial draft; M.R.: reviewing and revising; S.N.: writing and reviewing.

## Conflicts of interest disclosure

The authors declare no conflict of interest.

## Research registration unique identifying number (UIN)

1. Name of the registry: not applicable.
2. Unique identifying number or registration ID: not applicable.
3. Hyperlink to your specific registration (must be publicly accessible and will be checked): not applicable.

## Guarantor

Sardar Noman Qayyum.

## Data availability statement

Not applicable.

## Provenance and peer reviewed

Not commissioned, externally peer-reviewed.
